# Regulation to function: A computational approach to specialized metabolism

**DOI:** 10.1371/journal.pbio.3003205

**Published:** 2025-06-13

**Authors:** Justin R. Nodwell

**Affiliations:** Department of Biochemistry, University of Toronto, Toronto, Ontario, Canada

## Abstract

Despite a century of use in drug discovery, specialized metabolism continues to churn out astonishing discoveries. This primer explores a new study in PLOS Biology that uses a novel computational approach to uncover previously unknown functions of a compound produced by Streptomyces coelicolor.

## Teaser

Despite a century of use in drug discovery, specialized metabolism continues to churn out astonishing discoveries. A new study in *PLOS Biology* uses a novel computational approach to uncover previously unknown functions of a compound produced by *Streptomyces coelicolor*.

Specialized metabolites are produced primarily by bacteria and fungi and serve diverse functions that include scavenging environmental iron, quorum signaling, photoprotection, and, most famously, the poisoning of other microorganisms, hence their use as antibiotics. This enormous ensemble of biochemical pathways is arguably the most important source of medicines in history. The enzymes that make up these pathways are encoded in biosynthetic gene clusters (BGCs). A grand challenge in this field is distinguishing the new and interesting against the background of the knowns. To date, the most important solution to this challenge is the BGC prediction tool known as antiSMASH, developed by Medema and colleagues [1]. This tool enables genome and metagenome data to be queried for BGCs with ease and, along with subsequent generations such as BiG-SCAPE and CORASON, it has revolutionized this field. Nevertheless, we continue to see surprises that eluded this powerful technology [2].

One under-tapped resource is the large literature on the regulation of BGC expression in the streptomycetes, most importantly *Streptomyces coelicolor*, but also *S. griseus*, *S. venezuelae*, *S. avermitilis*, *S. clavuligerus,* and *S. virginiae*. In these well-studied specialized metabolizers, there is sufficient information to assemble regulatory networks of varying degrees of detail and sophistication. This body of knowledge is the result of decades of painstaking molecular research aimed at identifying important regulators of specialized metabolism and the promoter elements they recognize. Naturally, these promoter elements are enriched in the BGCs. A recent study by Augustijn and coworkers [3] takes an important step toward computationally leveraging this knowledge for the discovery of new specialized metabolites.

Their approach takes advantage of the idea that identifying genes controlled by regulators that have known roles can be used to predict the function of genes or gene clusters. There have previously been attempts to leverage regulation as a predictive tool in *Streptomyces* [4], however, these have not resulted in broadly applicable computational tools. In this case [3], the authors leveraged the extensive literature on transcription factors and their binding sites in the species *S. coelicolor.* They searched this genome for putative binding sites for transcription factors that have relatively well-understood physiological roles and identified genes and operons of unknown function. This involved parsing 730 putative binding sites for a number of relatively well-understood transcription factors, distinguishing the probable from the improbable, and correlating binding sites with antiSMASH results to identify the genes that had the greatest potential as novel specialized metabolites. Doing this work in *S. coelicolor* increased the challenge because, as the authors acknowledge at the outset, this species has the best characterized specialized metabolome, with 27 fully or partially characterized compounds and BGCs.

Augustijn and colleagues landed on an operon predicted to be controlled by DmdR1, a regulator of iron-related genes, which they named *desJGH* [3]. Using well-established chemical and biological approaches, they were able to show that the products of these genes mediate previously unknown steps in the biosynthesis of several desferrioxamines. This is surprising, because this family of iron siderophores has been extensively studied, including in *S. coelicolor*, and harnessed medically for the management of iron toxicity. That this computational tool, founded on models of gene regulation, was able to identify new features in this otherwise well-known pathway is striking evidence of the potential of this approach. And if such discoveries are hiding in plain sight in a well-studied species like *S. coelicolor*, surely there is more exotica to be found in the thousands of environmental isolates in collections around the world. Or, perhaps more pointedly, in the extraordinary genome and metagenome databases that are at our disposal. The time is right for a new approach.

This work [3] also raises some important questions. The first concerns the breadth of application of this approach. At present, *S. coelicolor* surpasses all other *Streptomyces* species in the extent to which its specialized metabolism is understood*.* What we do know, however, indicates that while the regulatory players might be conserved across species, some of their roles are not. An obvious example is the γ-butyrolactones, a conserved group of signaling molecules that regulate specialized metabolism in many species, but have varying roles in spore formation [5]. Another question concerns regulatory details that remain unknown in *S. coelicolor*. For example, the *absA* operon encodes a two-component system known to serve a repressive role for at least three important specialized metabolites in *S. coelicolor*. However, in spite of concerted efforts by three groups, the binding site of the AbsA2 response regulator is not known [6,7].

These questions focus our attention on issues that need to be addressed more broadly in the *Actinobacteria*, especially in the thousands of *Streptomyces* species*.* While the discovery of important specialized metabolites continues apace, the intensity of research on the regulation of biosynthetic genes has slowed in recent years. This new study [3] supports the argument that the time is ripe for fresh approaches to this question: understanding the conservation of regulatory strategies between species seems like a critical aim; linking more of the regulators to physiological and ecological outputs seems like another. The scope of these questions suggests that to address them we will need to lean heavily on computational approaches. The work of Augustijn and colleagues is an important step in that direction.

## Abbreviation

BGCs: biosynthetic gene clusters
